# A comprehensive analysis of *Trehalose-6-phosphate synthase* (*TPS*) gene for salinity tolerance in chickpea (*Cicer arietinum* L.)

**DOI:** 10.1038/s41598-022-20771-x

**Published:** 2022-09-29

**Authors:** Tapan Kumar, Neha Tiwari, C. Bharadwaj, Manish Roorkiwal, Sneha Priya Pappula Reddy, B. S. Patil, Sudhir Kumar, Aladdin Hamwieh, T. Vinutha, Shayla Bindra, Inderjit Singh, Afroz Alam, Sushil Kumar Chaturvedi, Yogesh Kumar, M. S. Nimmy, K. H. M. Siddique, Rajeev K. Varshney

**Affiliations:** 1 ICAR-Indian Agricultural Research Institute, Pusa, New Delhi 110012 India; 2 International Centre for Agricultural Research in the Dry Areas, Amlaha, Madhya Pradesh 466113 India; 3 Khalifa Center for Genetic Engineering and Biotechnology, United Arab Emirates University, Al-Ain, United Arab Emirates; 4 International Centre for Agricultural Research in the Dry Areas, 2 Port Said, Victoria Square, Maadi, Cairo, Egypt; 5 Punjab Agricultural University, Ludhiana, India; 6 Banathali Vidyapith, Banasthali, Rajasthan India; 7 RLBCAU, Jhansi, India; 8 ICAR-IIPR, Kanpur, India; 9 ICAR-NIPB, New Delhi, 110012 India; 10 The UWA Institute of Agriculture, UWA, Perth, WA Australia; 11 International Chair in Agriculture & Food Security, State Agricultural Biotechnology Center, Centre for Crop & Food Innovation, Food Futures Institute, Murdoch University, Perth, Australia

**Keywords:** Molecular biology, Plant sciences

## Abstract

Soil salinity affects various crop cultivation but legumes are the most sensitive to salinity. Osmotic stress is the first stage of salinity stress caused by excess salts in the soil on plants which adversely affects the growth instantly. The Trehalose-6-phosphate synthase (*TPS*) genes play a key role in the regulation of abiotic stresses resistance from the high expression of different isoform. Selected genotypes were evaluated to estimate for salt tolerance as well as genetic variability at morphological and molecular level. Allelic variations were identified in some of the selected genotypes for the *TPS* gene. A comprehensive analysis of the *TP*S gene from selected genotypes was conducted. Presence of significant genetic variability among the genotypes was found for salinity tolerance. This is the first report of allelic variation of *TPS* gene from chickpea and results indicates that the SNPs present in these conserved regions may contribute largely to functional distinction. The nucleotide sequence analysis suggests that the *TPS* gene sequences were found to be conserved among the genotypes. Some selected genotypes were evaluated to estimate for salt tolerance as well as for comparative analysis of physiological, molecular and allelic variability for salt responsive gene Trehalose-6-Phosphate Synthase through sequence similarity. Allelic variations were identified in some selected genotypes for the *TPS* gene. It is found that Pusa362, Pusa1103, and IG5856 are the most salt-tolerant lines and the results indicates that the identified genotypes can be used as a reliable donor for the chickpea improvement programs for salinity tolerance.

## Introduction

Chickpea (*Cicer arietinum* L.) is a diploid plant with 2n = 16 chromosomes. The genome size is approximately ~ 738 mega-base-pair, with an expected 28,269 genes^1^. Chickpea is grown mainly as a rain-fed crop on residual soil moisture after the rainy season without or restricted irrigation. Abiotic stress viz. drought, heat and salt stresses are the major constraints to its production. Chickpea is one of the most important food legumes, grown in various regions and climatic conditions^2^. Globally it is cultivated in the area of 13.72 million hectares (Mha) and the annual production is recorded as 14.25 million tons (Mt)^3^.

Among the legumes fababean, chickpea, and field pea are more sensitive to salinity^4^. Salt-affected soils are present in the independent borders of approximately 75 countries and covering more than 20% of the global irrigated area^5,6^. Salinity-related yield losses are estimated to be around 8–10 percent of total world production^7^. Osmotic stress is the first stage of salinity stress caused by excess salts in the soil on plants which adversely affects the growth instantly. Almost 20% of irrigated land is salt-affected, which is one-third area of food production worldwide^8,9^. With the passage of time, this percentage is rising^10^. Increasing artificial irrigation and over-irrigation worldwide suggests that, 50% of all arable land will be salinized by the year 2050^11^. Soil salinity also affects the plant germination and therefore the crop establishes very poorly, as a result, all further growth stages of the crop are affected simultaneously^12–14^. Salinity is major abiotic stress after drought, which affects crop production in various parts of the world^15^. Salt tolerant genotypes are able to maintain high shoot biomass and yield under salinity^16,17^. Over eight hundred million hectares of land is salt-affected and over 434 million ha suffer from an associated condition of sodicity^8^.

Salt tolerance is the capability of a plant to grow in saline soils and give yield normally without major loss. Soil salinity is a natural property of soil and therefore no avoidance or escape of saline conditions possible. Hence, soil salinity does not show seasonal variation and is difficult to manage. In this view, the mechanisms of salinity tolerance cannot be classified into the stress escape and stress avoidance, as explained for drought stress. The plant can manages the salt stress at the three levels i.e. at a whole plant level, the cellular level of the plant and the molecular level of the plant^18^.

Different biochemical analyses such as mutational analysis or analysis of linkage for the interested trait are the criteria of selection and identification of candidate genes^19^. Improved sequencing technology facilitates the quick and low cost method through which enormous sequence data can be generated and eventually helpful for the identification of genes responsible for stress tolerance. Candidate genes which are responsible for abiotic stress can be identified by the use of biotechnological approach, and further can be used in crop management or improvement.

Trehalose is an alpha, alpha-1, 1-linked glucose disaccharide. Trehalose is present in very low amounts in angiosperms and it is found that in abiotic stresses moderately increase of trehalose enzyme in plant^20–22^. Trehalase can function as an osmolytes at a threshold level and stabilize the membranes and proteins of plant^23^. Trehalose-6-phosphate (T6P) generated by Trehalose-6-phosphate synthase (*TPS*) from glucose-6-phosphate and UDP-glucose followed by dephosphorylation to trehalose and trehalose-6-phosphate phosphatase (TPP) after that trehalase breaks down trehalose into glucose molecules^23^. Trehalose-6-phosphate synthase (*TPS*) is one of the important enzyme genes involved in trehalose biosynthesis, which provides protection against salt stress^24^. Trehalose metabolism is positively regulated in abiotic stress tolerance. Gene expression responsible for the trehalose pathway confirms that drought and salt stress tolerance increased in several plant types^25^. Resistance to drought, salinity and cold was observed from the high expression of different isoforms of *TPS* in rice^26^. In Arabidopsis plants the overexpression of trehalose increased tolerance to drought stress and its plays an important role in the regulation of stomatal closure during drought stress^27^.

To identify the suitable genotype, the present investigation of diverse collections of chickpea were taken for this research for physiological, molecular and identification of the gene for salt tolerance.

## Results

### Comparative performance of the genotypes

**Figure 1:**
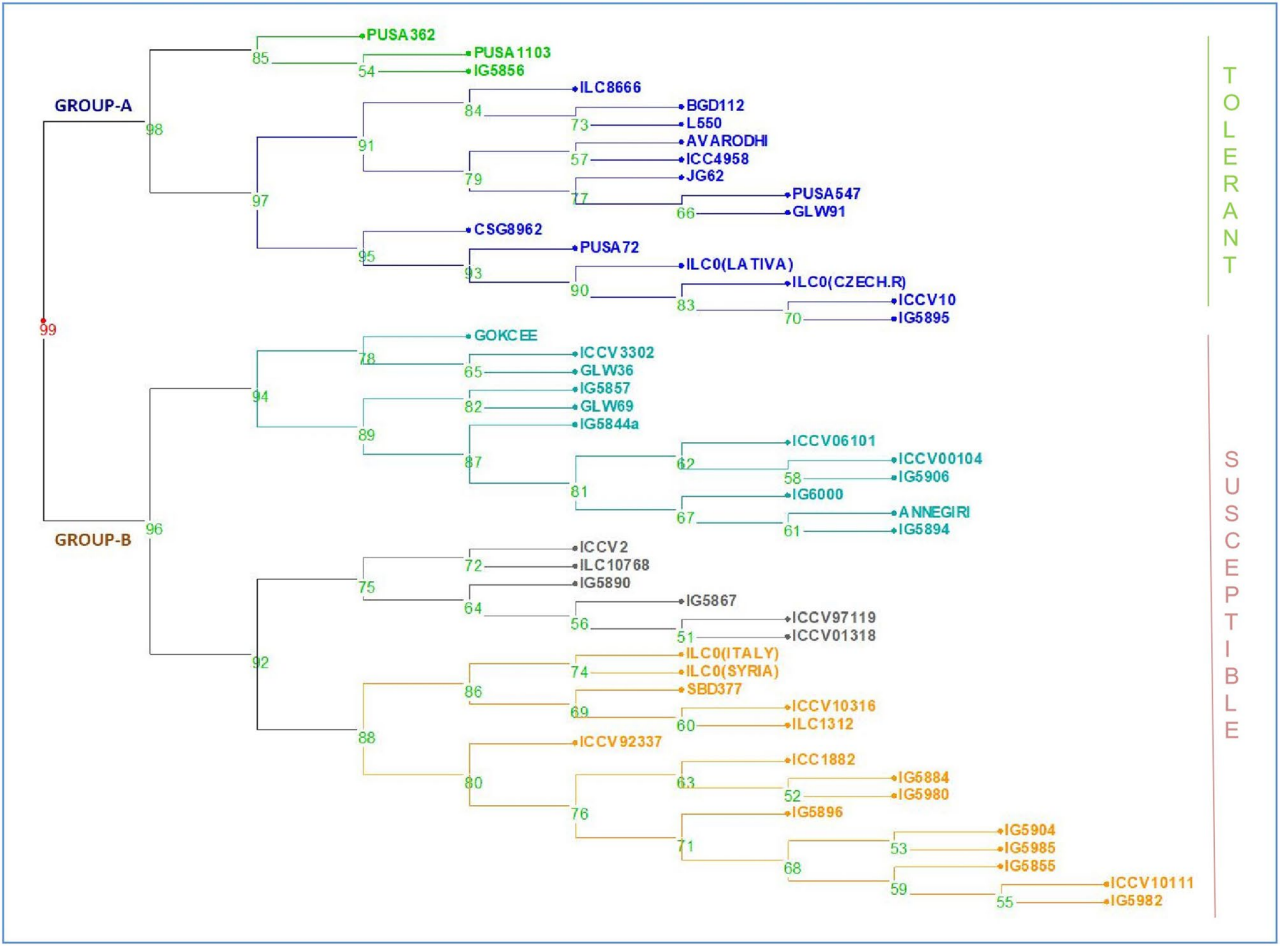
Dendrogram generated from an unweighted pair group method analysis (UPGMA) cluster analysis based on all the stressed morphological characters for salt stress. Tolerant genotypes were sub-grouped into highly tolerant to moderately tolerant. Similarly, the susceptible genotypes were grouped and sub-grouped.

A significant variation was detected for control and salt treatments for most of the investigated traits. There were decreases in means values for maximum traits in saline condition (Table 1). Analysis of variance (two-way) was done for all the traits under control and salt stress conditions. Significant variability was found among the genotypes for the mean sum of the square for all the traits (Table 2). Euclidean distances were calculated for the genotypes understudy. The genotypes were groups according to their tolerant or susceptible traits. Tolerant genotypes were sub-grouped into highly tolerant to moderately tolerant. Similarly, the susceptible genotypes were grouped and sub-grouped (Fig. 1). A 2D plot was generated by all the morpho-physiological data for the estimation of genetic variation in the genotypes. Scattered plot revealed a pattern of mostly two groups which were distinctively separated the tolerant and susceptible genotypes (Fig. S1).

**Table 1:** A significant variation was detected for control and salt treatments for most of the investigated traits.

Traits	Normal					Salt stress					Decrease (%)
	Mean ± SE	CV (%)	Range	h^2^	GA	Mean ± SE	CV (%)	Range	h^2^	GA	
DTF	69 ± 1.64	7.12	46–97	0.959	34.544	74 ± 1.58	4.58	53–103	0.956	33.062	**− **8.15
DTM	135 ± 0.45	2.38	126–139	0.586	4.667	109 ± 0.56	6.59	87–137	0.634	4.911	18.93
PH	53 ± 1.28	7.02	37–78	0.959	15.772	18 ± 0.90	5.56	11–33	0.200	2.014	66.53
PPP	38 ± 0.87	6.15	22–47	0.718	17.368	7 ± 0.60	9.05	3–24	0.480	3.838	81.28
SPP	2 ± 0.07	10.93	1–2	0.627	1.699	1 ± 0.06	8.88	1–2	0.488	1.026	13.33
RWC	61 ± 1.56	7.94	46–83	0.766	7.805	61 ± 1.77	8.67	43–86	0.597	4.788	1.36
MSI	60 ± 1.69	9.87	44–87	0.808	13.796	57 ± 1.74	11.69	40–82	0.470	3.949	5.86
YLD	34 ± 1.07	12.05	24–58	0.921	19.211	9 ± 0.75	11.74	2–23	0.884	14.217	74.86

*DTF* Days to 50% flowering, *MSI* Membrane Stability Index, *DTM* days to maturity, *PH* Plant Height, *PPP* Pods per Plant, *SPP* Seeds per Pod, *RWC* Relative Water Content, *YLD* Plant Yield.

**Table 2:** Analysis of variance (two-way) was done for all the traits under control and salt stress conditions.

Source of variation	Mean sum of square							
	DTF	MSI	DTM	PH	PPP	RWC	SPP	YLD
Genotype	1994.73**	849.51**	579.72**	140.56**	109.13**	802.64**	1.04**	204.50**
Treatment	2352**	936.33**	48,615.87**	93,598.003**	72,168.03**	52.92**	3.41**	49,295.46**
Genotype Treatment	50.00**	28.28**	432.80**	224.86**	58.091**	30.51**	0.17**	51.45**
Residual	2.363	4.376	2.35	3.829	2.43	1.01	0.05956	2.335

*^,^**Is the significance at 5% and 1% respectively. *DTF* Days to 50% flowering, *MSI* Membrane Stability Index, *DTM* Days to Maturity, *PH* Plant Height, *PPP* Pods per Plant, *RWC* Relative Water Content, *SPP* Seeds per Pod, *YLD* Plant Yield.

Pearson’s correlation analysis examined the relationship between traits and seed yield. Maturity days were significantly correlated with the flowering days, height of the plants was positively correlated with maturity days, negative correlation was found between seed per pod is showing with days to flowering. Plant yield was positively correlated to days to maturity (r = 0.601), membrane stability index (r = 0.410), plant height (= 0.810), pods per plant (0.884), relative water content (r = 0.326) and seeds per pod (0.354) (Table 3). Broad sense heritability and genetic advance were analysed under normal and saline condition. Moderate heritability was found with low genetic advance for plant height, pods per plant, relative water content, and membrane stability index under saline condition. Genotypes which have retained, sufficient plant height, pods per plant with water retention capacity, and membrane stability index under the saline condition are possible to give good production in the saline condition. (Table 1).

**Table 3:** Pearson’s correlation analysis examined the relationship between traits and seed yield.

	DTF	DTM	MSI	PH	PPP	RWC	SPP	YLD
DTF	1**							
DTM	0.393**	1**						
MSI	0.01	0.118	1**					
PH	− 0.021	0.679**	0.161	1**				
PPP	− 0.183	0.648**	0.271**	0.885**	1**			
RWC	− 0.034	0.027	0.902**	0.05	0.16	1**		
SPP	− 0.231**	0.098	0.384**	0.185	0.268**	0.448**	1**	
YLD	− 0.157	0.601**	0.41**	0.81**	0.884**	0.326**	0.354**	1**

*DTF* Days to 50% Flowering, *DTM* Days to Maturity, *MSI* Membrane Stability Index, *PH* Plant Height, *PPP* Pod Per Plant, *RWC* Relative Water Content, *SPP* Seeds per Pod, *YLD* Plant Yield. **Indicates significant at 1%  level of significance.

### Sequence similarity and allelic variation of *TPS* gene

**Figure 2:**
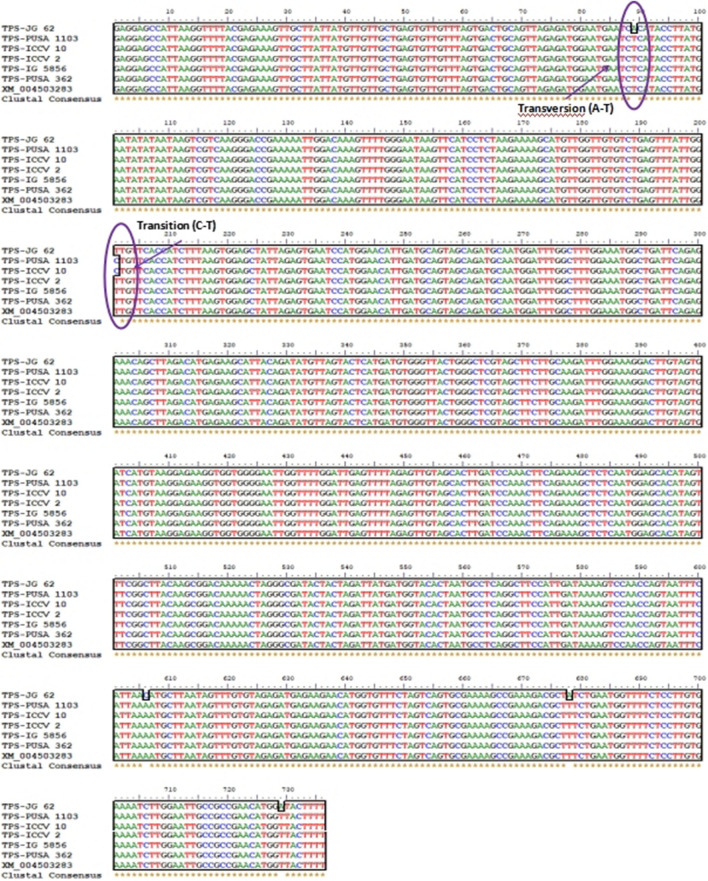
Multiple sequence alignments analysis using BioEdit (software package) of the nucleotide sequences. The rectangular box shows a total of five SNPs (1 Transition and 4 Transversion) were detected in the sequence of the TPS gene.

Based on physiological data six genotypes were selected for allelic variation through sequencing. Trehalose-6-Phosphate Synthase (*TPS*) gene homolog was amplified using the gene-specific primers. The size of amplicons was ranged from 740 to 821 bp in length (Fig. S2). The results show the highest identity with the homologous *Cicer arietinum TPS* gene (XM_004503283). The identified gene sequences were submitted in NCBI and the following IDs were provided: JG62 (MF503402), PUSA1103 (MF503403), ICCV10 (MF503405), ICCV2 (MF503406), IG5856 (MF503407), and PUSA362 (KY542279) (Table S2). The gene sequence from the PUSA1103 genotype has shown the highest identity (99%) with e-value 5e-156 with the *TPS* gene (XM_004503283) (Table S3). Nucleotide diversity was not observed for the *TPS* gene. Finding positions of SNPs in nucleotide sequences was done manually by aligning the sequences in BioEdit software. A total of five SNPs (1 Transition and 4 Transversion) were detected in the sequence of the *TPS* gene (Fig. 2). The SNPs were found in the genotypes of PUSA1103, ICCV10, and JG62. No indels were observed across the *TPS* gene sequences.

### Structural features of the gene

**Figure 3:**
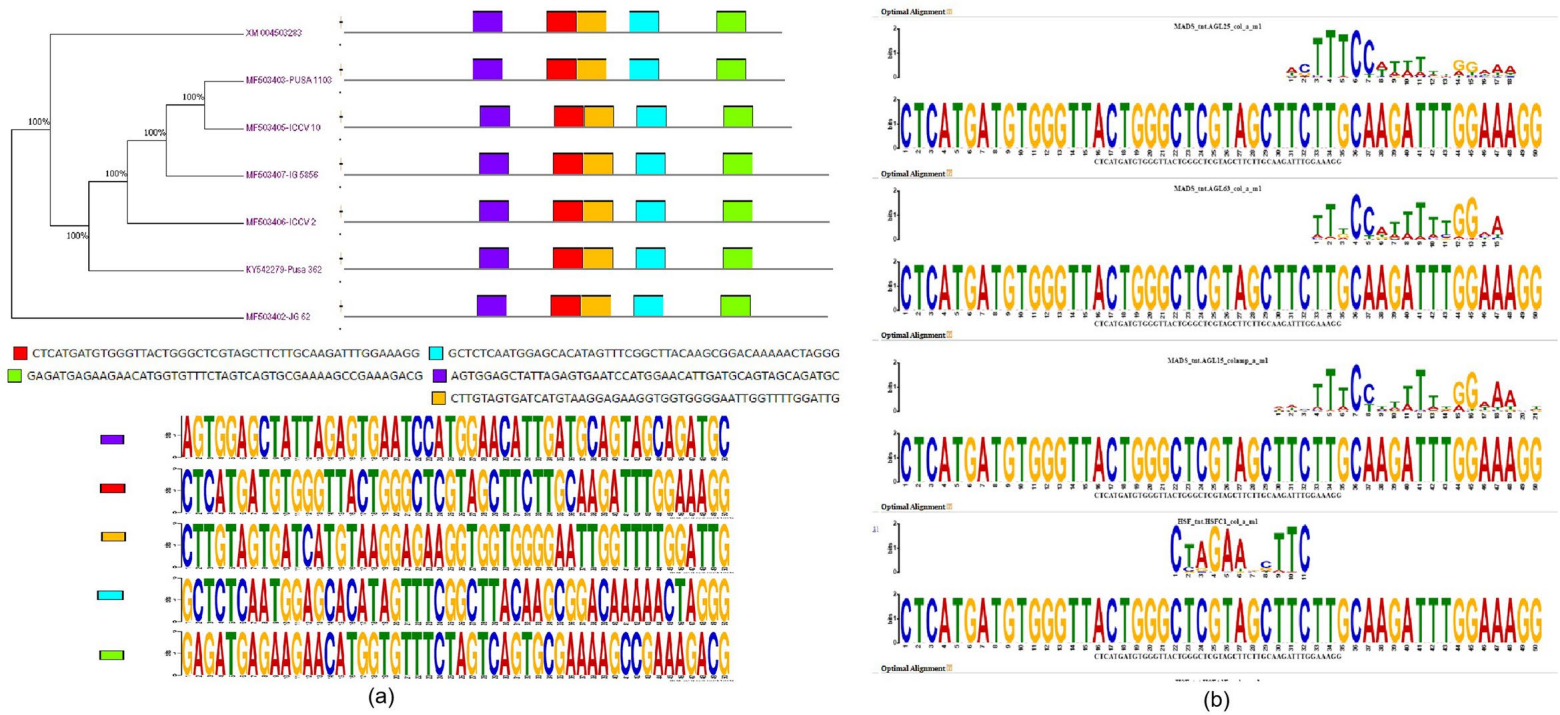
Motifs prediction using MEME motif search program. (**a**) predicting motif patterns of *TPS* gene and their phylogenetic relationships among the genes. Different motifs are represented by different colour boxes with motif sequences. The genotypes have five motifs starting from 217 to 675 nt length (**b**) The alignment of predicted motifs obtained via MEME/TOMTOM tool and WebLogo plot of consensus motifs in each TPS gene.

Candidate motifs were compared using the TOMTOM tool with sets of motifs from Arabidopsis^30^. Most of the TOMTOM hits results from searching a single DNA motif show that the query motif closely resembles the binding motif for orthologous transcription factor proteins in Arabidopsis (Table S4). These prime sequences and their similar transcription factors suggest that our results may have identified mitigating follow-up studies. Motifs prediction using MEME motif search found that all the *TPS* genes from different genotypes have five motifs starting from 217 to 675 nt. (Fig. 3a). The predicted motifs were further analyzed by Tomtom (a motif comparison tool from MEME-SUIT) to identify motif-specific potential transcription factors. The results suggest that the five motifs were identified as conserved motifs (Fig. 3b).

Motif 1: Minichromosome maintenance1 Agamous Deficiens Serum response factors-MADS-box TFs AGL25 (AGAMOUS-Like25:involved in seed germination by influencing the ABA catabolic pathway and regulation ABA signaling), AGL63 (AGAMOUS-Like63: expressed in seeds and embryos; growth and Transcription regulation), AGL13 (AGAMOUS-Like13; highly expressed in Lateral root cap); (CBF4): involved in cold and drought tolerance, responsive to ABA and regulate drought adaption); Motif 2: ARF2 (Auxin response factor 2) transcription factor, plays an important role in auxin signaling, plant growth, development, and stress response. ARF2 may affect seed size and drought tolerance through regulating ABA signaling, Cys2/His2 (C2H2) zinc finger proteins play an important role in abiotic stress-resistant plants. Basic leucine zipper (bZIP52) gene-regulates metabolic reprogramming during stress. Motif 3: RWP-RK a putative DNA-binding domain, which was previously proposed in the primary structure of NIN, NIN-LIKE PROTEIN genes (NLP7) is a regulatory protein associated with nitrogen assimilation, WRKY50-a DNA binding proteins by the presence of the peptide sequence (Trp-Arg-Lys-Tyr) followed by a Zn-finger domain. They are important for stress-induced transcriptional reprogramming, NAC (ANAC094): NAC domain-containing protein 94 functions in transcription factor activity and is involved in the regulation of transcription. Motif 4: interacted with NLP (AtNLP4), bZIP (bZIP28), HSF. Motif 5: MYB (MYB96 and MYB 94); these transcription factors have been found to be involved in the drought response. HMG (High mobility group)-AT4G11080 (3xHMG-box1) proteins are abundant chromatin-associated proteins found in nuclei that interact with mitotic and meiotic chromosomes.

Isoelectric point (PI) ranged from 7.12 (MF 503407_IG 5856) to 8.22 (MF503403_PUSA 1103). The protein weight ranged from 28.11 KDa (MF503403_PUSA 1103) to 31.27 KDa (KY542279_Pusa 362). The instability index of 43.97 indicates the instability of the *TPS* protein. The grand average of hydropathicity (GRAVY) of protein represents the negative value ranging from − 0.132 (MF503405_ICCV 10) to − 0.247 (MF503402_JG 62), indicates the non-polar nature of the protein. The conserved domains in *TPS* protein were identified with two superfamilies: Glycosyltransferase family 20 (pfam00982; E-value 1.43e−27 and Trehalose-phosphatase (pfam02358; E-value: 7.12e−18).

**Figure 4:**
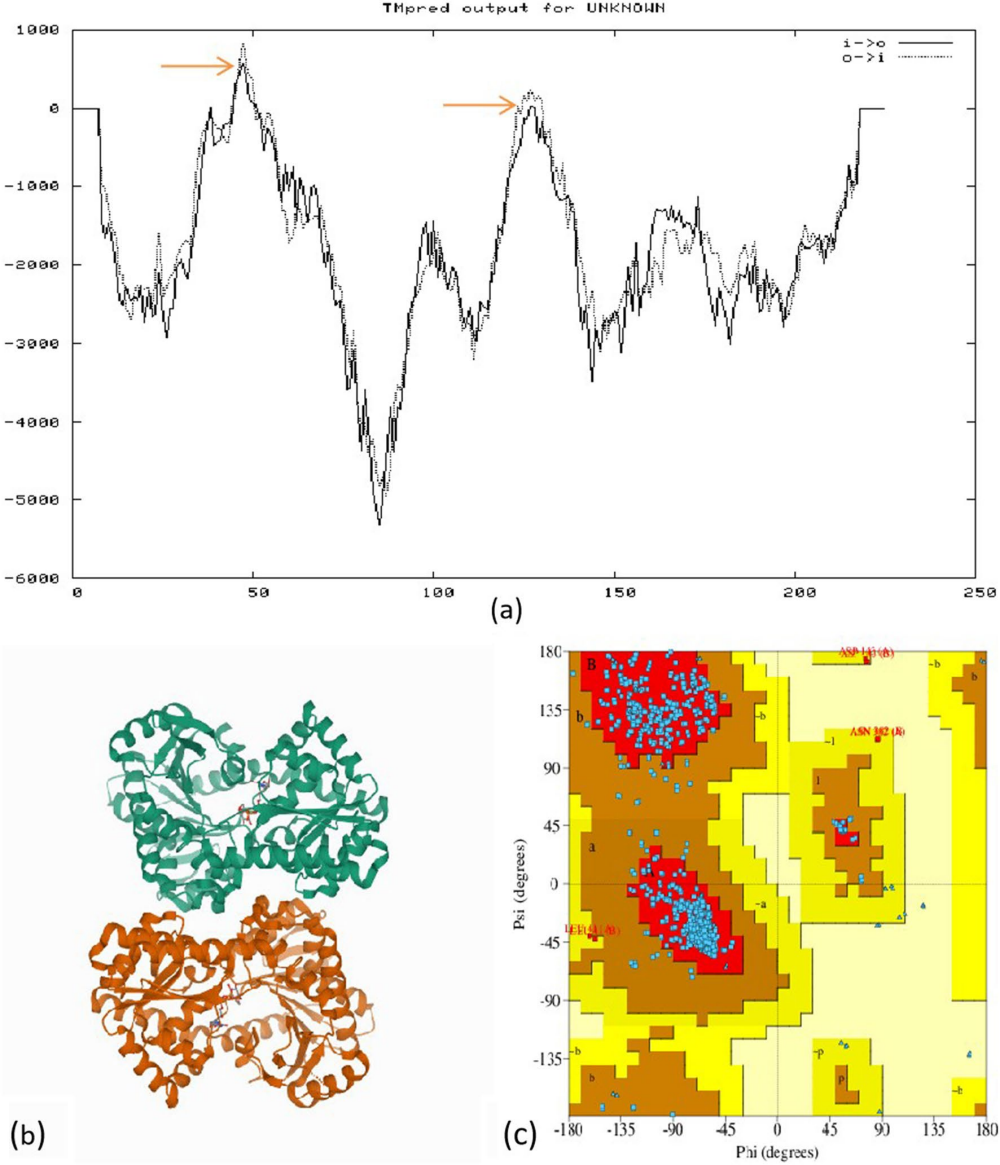
(**a**) Trans-membrane analysis of the *TPS* protein done by TMpred software. The X axis represents the TPS protein lenght from N-to C-terminal and Y axis shows the score computed by the program. The main hydrophobic areas in the *TPS* protein are indicated by the arrow. (**b**) Protein 3D structure helps to visualize the protein positional features of TPS developed by PDBsum, where green colored represent A chain, red colored represent B chain and yellow colored in the center represent ligand. (**c**) Structure prediction of *TPS* domain containing protein (ii) PROCHECK was used to measure the accuracy of the modeled protein by Ramachandran plot. The 3D model generated is also supported as around 99% of residues are present in favored and allowed regions.

**Figure 5:**
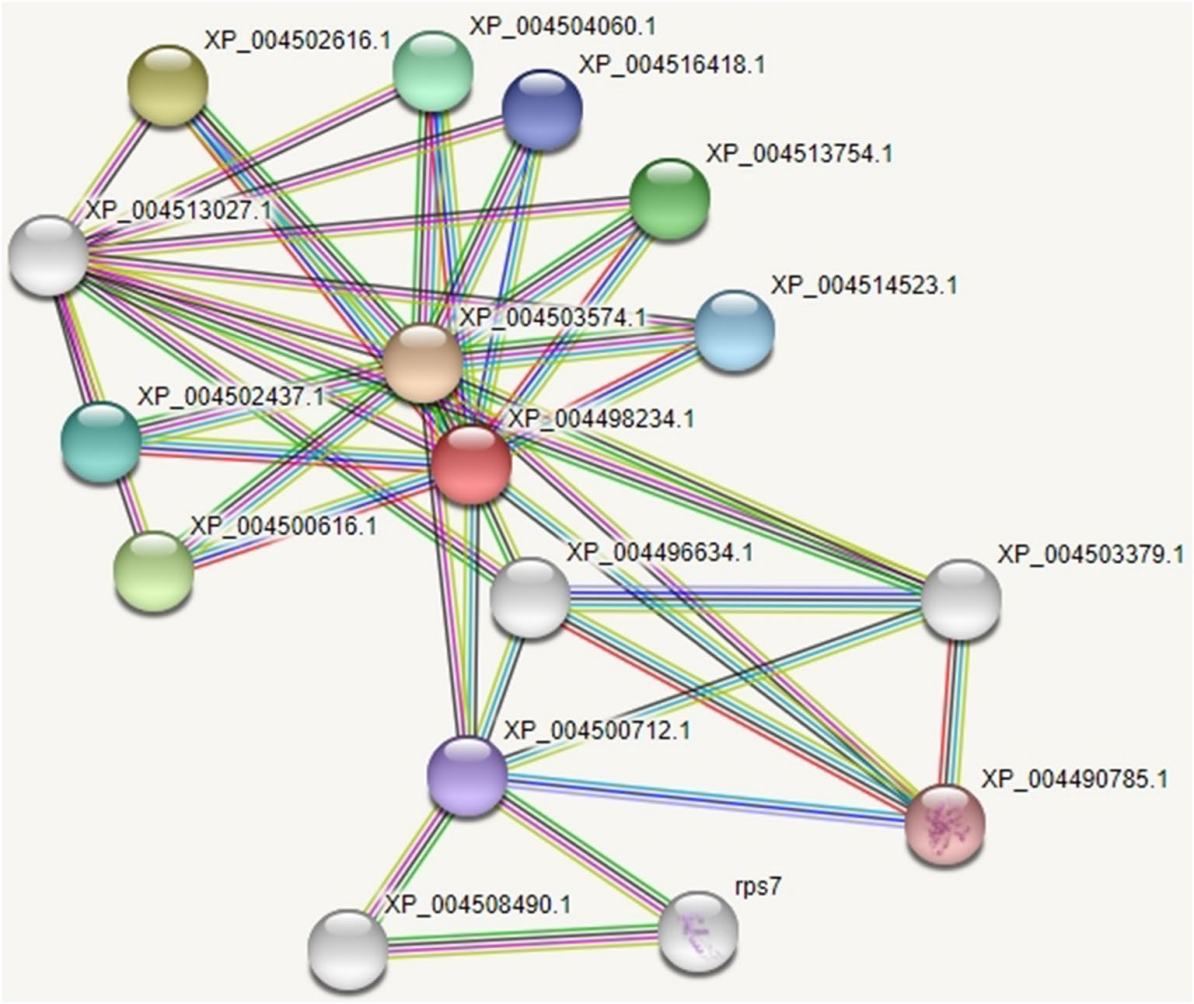
STRING network analysis for protein–protein interactions (PPI) of the target protein trehalose phosphate synthase. The PPI network shows that the target protein (*TPS*) interacts with a range of proteins. The nodes in the network represent the protein and the links are the interaction between the protein.

The transmembrane analysis using the TMpred tool revealed two strong trans membrane helices with a score of 820 and 574. However, lower than 500 scores did not support it to consider as a transmembrane protein. The main hydrophobic areas in the *TPS* protein are indicated by the arrow (Fig. 4a). The three-dimensional structure of a protein can be predicted from amino acid sequences by a web-based homology modeling tool at different levels of complexity. The 3D model of the *TPS* protein was constructed by using the RCSB/PDB: Protein database. The protein was found to have alpha helixes (44.3%), β sheet (18.6%), 310-helix (3.4%), and others 33.7% of the total target protein (Fig. 4b). The Psi-phi plot revealed that 91.6% amino acids along with 760 residues were found in the most favored regions, 7.7% of amino acids along with 64 residues were found in the allowed regions, and no residues were found in the disallowed regions (Fig. 4c). The protein–protein interaction (PPI) network of the hypothetical protein Trehalose-6-Phosphate Synthase (*TPS*) was obtained by using the STRING database (Fig. 5).

Our results suggest that in order to carry out its functions, the target protein (*TPS*) interacts with a range of proteins. The interacting proteins are XP_004503574.1 Trehalase (582 amino acid), XP_004502616.1 Trehalose 6-phosphate phosphatase (388 amino acid), X P_004516418.1 Trehalose-phosphate phosphatase 1-like (266 amino acid), XP_00 4500712.1 UTP-glucose-1-phosphate uridylyltransferase-like (629 amino acid), XP_0 04490785.1 UTP-glucose-1-phosphate uridylyltransferase (470 amino acid), XP_0045 13027.1 PP2Cc-Protein phosphatase 2C and cyclic nucleotide-binding/kinase domain-containing protein (1078 amino acid), XP_004503 379.1 Phosphoglucomutase, cytoplasmic-like isoform X1; belongs to the phosphohexose mutase family (582 amino acid), XP_004508490.1 The predicted functional partners interacting with *TPS* confirms that it may have a role in salt stress tolerance in chickpea.

## Discussion

There is an unfavourable physical environment that causes abiotic stresses like drought, salinity, heat, and chilling^11,31^. The salinity of soil is determined by the concentration of the soluble salt (Ece is > 4 dS/m). Due to salt stress the root and shoot growth is impaired and if the concentration is high for a long time then the plant death as a result^18^. Salt tolerance is the capability of a plant to grow in saline soils and give yield normally without major loss. Soil salinity is a natural property of soil and therefore no avoidance or escape of saline conditions is possible.

Chickpea plants can be affected by 25 mM NaCl in the experiments^7^. Allele mining based on sequencing involves identifying the nucleotide variations of polymerase chain reaction-based amplified alleles of a gene in the specific genotypes. A number of alleles can be identified among the cultivars through this approach. It allows recognizing the effect of mutation insertions or deletions (InDels) in the gene structure and point mutations and to construct haplotypes. Two-way ANOVA results disclosed the presence of significant variations in the genotypes understudy for plant height, days to flowering, days to maturity, seeds per pod, 100 seed weight, plant yield, RWC, and MSI. Salinity stress lowers the seed yield of all genotypes based on the tolerance capacity varied from genotype to genotype. There were differences among the genotypes in yield under normal and saline condition seeds per pod and 100 seed weight were less affected due to the additive nature of genes controlling this trait. This has been found that salt stress delayed flowering and the delay was more in the sensitive genotypes than the tolerant genotypes. The number of filled pods and the number of seeds per plant were associated with seed yield under salt stress. Under salt stress, the weakened reproductive machinery is more in sensitive than the tolerant lines^32^.

There are various assumptions regarding the ideal selection conditions for acquiring stress-tolerant genotypes for the usage of targeted environment based on heredity and expected genetic advance^33,34^. Because of the increased heritability and anticipated genetic advance from selection for grain yield, some researchers found that the non-stressed environment selection is better than the stressed environment^35,36^. On the other hand, stressed environments are also determined to be superior to non-stressed environments due to their increased heritability and higher genetic gain via selection^37,38^.

Putative candidate genes of trehalose-6-phosphate synthase (*TPS*) were isolated using particular sequence information got from the *TPS* gene sequences available in NCBI from thirteen genotypes. The results indicated that the trehalose-phosphate synthase primers designed were specific to the single region in the chickpea genome. The sequence similarity analysis shows the highest identity with the homologous *TPS* gene. The trehalose-phosphate synthase gene was found conserved among the genotypes and this suggests that the primers designed were accurate for the desired gene. A comprehensive analysis was done for the *TPS* gene from the selected genotypes of in the present study. The nucleotide sequence analysis suggests that the *TPS* gene sequences were found to be conserved among the genotypes. The amino acid sequences of the *TPS* gene share highest homology with the reference (XM_004503283) *TPS* gene.

Although, the sequence of the six *TPS*s genes showed high similarity among them their gene lengths were slightly differentiated. The sequence similarity analysis disclosed that the homozygous alleles of SNPs are found in position 201 in the genotype Pusa1103 and a salt-tolerant genotype ICCV10. In addition, three SNPs are found in genotype JG62. This is the first report of allelic variation of *TPS* gene and results indicate that the genotype Pusa1103 with the SNP has also shown a significantly higher value of RWC, MSI, and yield in comparison to other genotypes and this suggests that the SNPs present in these conserved regions may contribute largely to functional distinction.

The fact that our analyses identified five motifs failing into different unique putative promoter features that await experimental verification is especially significant. Three of the five specific motifs found are supported by similar motifs, and one validates an earlier computational prediction made in other plant species using a different approach^39^. The predicted motifs found through using motif search for the given sequences have interacted with regulatory proteins. Regulatory proteins have an important function in signal transduction by inducing the expression of target genes. Transcription factors (TFs) bind to the promoters of their target genes and regulating gene expression which affects the phenotype^40^. Abiotic stress signaling pathways are regulated by transcriptional factors^41,42^. It is found that C2H2 transcriptional factors act as transcriptional activators or can repress the regulation of gene to salt stress responses^43^. Also, C2H2-type zinc finger proteins can improve plant salt tolerance by maintaining ionic balance. Salt-tolerance-related zinc finger proteins (STZ) enhance salt tolerance by regulating the expression of ionic balance-related genes^44^. The gene OsbZIP71 (bZIP: Basic leucine zipper) transcription factor activates ABA-responsive gene expression to increase the salinity and drought tolerance in rice^45,46^. A recent study supports our findings of motifs predictions suggests that the isomers of trehalose can mediate ABA-induced stomatal closure during drought stress and the isomer localized in cytoplasm/nuclear due to lack transmembrane domain are more effective in stress conditions^47^.

In the present study, the physio-chemical characterization was also performed by computing theoretical isoelectric point (pI), molecular weight, instability index, and GRAVY of the *TPS* protein. The result suggests that most of the *TPS* proteins have more hydrophilic areas than hydrophobic areas, which is in accordance with the value of the GRAVY. So, the *TPS* protein is hydrophilic in nature with good solubility. The transmembrane analysis suggests that both inside to outside and outside to inside helices were distributed in areas 39 to 56. The transmembrane proteins may act as the main functional protein under stress. Tamura et al.^48^ reported that NtC7 a new type of transmembrane protein that belongs to the receptor-like protein family also responded to the salt and osmotic stresses^48^.

The analysis of secondary structure suggests that the higher percentage of α-helices in the structure may be responsible for increasing the hydrophilic properties of the protein. As in some of the studies, it was found that the surface hydrophobicity of protein increased with β-sheet content^49^. The three-dimensional structure offers valuable insights into the molecular function and in putative site identification of the studied protein. The results obtained were further validated by using the PDBsum tool by constructing the Ramachandran plot.

The triangle represents the glycine (60) and proline (40) residues, and the shading on the diagram shows the different regions, as defined by Morris et al.^50^. The darkest portions correspond to the "core" regions in the plot, which indicate the most favorable combinations of phi-psi values. The 3D model generated is also supported as around 99% of residues are present in favored and allowed regions. The predicted PPI network of *TPS* suggested that it has interacted with its catalytic partners (Trehalase-*TPS*/TPP) and also interacted with protein phosphatase 2C family, Phosphoglucomutase, and 30S ribosomal protein S7.

Previous studies have indicated that the genes of the protein phosphatase 2C family and a bZIP (transcription factor) play a crucial role as ABA-responsive element binding factors in the ABA signaling pathway^51^. Thus our results suggest that it may trigger a signal transduction pathway that involves calcium and ROS-mediated signaling and this may contribute to stress tolerance in plants. The trehalose contents are possibly modulated by trehalose-6-phosphate synthase (*TPS*) and play an important role in the trehalose biosynthetic pathway. Osmotic stress is the first stage of salinity stress caused by excess salts in the soil on plants which adversely affects plant growth instantly^52,53^. A plant cell adjusts its osmotic adjustments by the accumulation of companionable osmolytes to manage the osmotic stress. An experiment on rice has revealed that under salinity stress cells accumulate trehalose and also decrease sodium accumulation, salT (an osmotically regulated gene) expression, and growth reduction in plants^54^. Another study on rice suggests that the accumulation of trehalose in plants gives increased tolerance to abiotic stresses^20^.

Gene expression responsible for the trehalose pathway confirms that salt stress tolerance increased in several plant types^25^. Resistance to salinity was observed from the high expression of different isoforms of *TPS* in rice^26^. The sequenced data generated can be further used in crop improvement programs by SNPs related to the preferred trait by the transgenic approach. It will also be beneficial to identify and validate genes that actually give insulation to these stresses so that they can be used as tools by breeders for the rapid identification of tolerant genotypes and for use in molecular breeding programs.

The identified genotypes can be used for the improvement of genetic and molecular breeding programs of essential traits through hybridizations. Identification of *TPS* gene and their allelic variations for the gene can fetch genomic resources with diverse alleles to develop better genotypes for salt tolerance. This study provides the identifications of promising genotypes for salt tolerance and candidate genes for better understanding of the molecular mechanisms of salt tolerance.

## Methods

Fifty diverse genotypes containing varieties, germplasm collection, landrace, and wild derivatives were taken from the collection of Chickpea Breeding Unit, Pulse Research Laboratory, Division of Genetics at ICAR-Indian Agricultural Research Institute, New Delhi (Table S1).

### Screening for salt tolerance

Screening to find out the suitable salt-tolerant genotype was done at the National Phytotron Facility of ICAR-Indian Agricultural Research Institute, New Delhi, during 2015–2016 and 2016–2017 under controlled greenhouse conditions as follows: The genotypes were grown in normal soil and saline soil conditions. 13 cm diameter pots were used which contain 6.5 kg of soil, with initial electric conductivity = 0.4 ds/m, pH 8.1, and were fertilized with 2 g of di-ammonium phosphate (DAP) for each pot.

The salt stress was given by treating an 80 mM solution of NaCl to the normal soil with an adequate volume to damp the soil to field capacity and saturated the whole pot soil. To fully saturate each pot and bring it to field capacity, approximately 1.50 L of the solution is required per pot^16^. Salt stress treatment was applied five days before the sowing. After sowing, the pots were watered with normal water to maintain the field capacity and to restrict the increase of salt concentration of the soil. The EC (Electric Conductivity) of individual pots was monitored weekly with the help of a conductivity meter. The EC of approximately 7.5–8.5 ds/m was maintained for each pot by further adding the required solution. The control pots (without salt) were initially watered with normal water with the required volume to reach the field capacity. Four seeds were sown in the control and salt treatment pots and when the plants germinated well two plants were removed. Two replicates for the control and treated pots were maintained and the mean values were used in all the analyses.

### Morphological characterization by agronomic data

Days to flowering (DTF), Days to maturity (DTM), 100 seed weight, yield per plant, Membrane stability index (MSI), and Relative water content (RWC) was the six parameters studied and data recorded^28^. The CROP-STAT (version 8.5) statistics tool was used to analyse the mean values of the samples from each replication. GenSTAT version 16.1 was used to generate a Pearson's Correlation matrix between the traits under control and saline conditions. The factorial and clusters analysis based on salt stress morphological traits was done by using DARwin 5 software 5.0.158.

### Identification of candidate gene for the salt stress tolerance

Based on and morpho-physiological data obtained from the genotypes, a subset of tolerant genotypes with positive and negative control was selected for the validation of markers linked to candidate genes (Table S3) associated with salinity stress. The PCR was done with selected genotypes to amplify the desired gene and purified products were selected for sequencing (Chromous Biotech Pvt. Ltd., Bangalore, India). The sequence analysis and nucleotide identity searches were done by BLAST at NCBI (www.ncbi.nlm.nih).

### Gel extraction of DNA fragment

The PCR was done to amplify the desired gene and amplified and purified products were selected for sequencing (Chromous Biotech Pvt. Ltd., Bangalore, India). The PCR products were electrophoresed on 1% agarose gel. From the gel, the expected bands were cut and scooped out. The agarose gel was purified according to the protocol of gel extraction kit (PureLinkTM Quick gel extraction kit, Invitrogen, Carlsbad, CA). The scooped gel was further dissolved by heating to 50 °C for 10 min in the solubilizing buffer by weight (w/v) of gel. The dissolved solutions were transferred into a Pure Link TM Clean-up spin column and spun at 10,000 rpm for 1 min. The flow-through were discarded and the columns were washed with washing buffer. The purified DNA obtained by eluted with 50 µL of elution buffer. The quality and quantity was rechecked by electrophoresis then stored at − 20 °C for the further uses of the DNA.

### Sequencing and analysis of the purified product

Trehalose-6-Phosphate Synthase (*TPS*) gene homolog was amplified using the gene-specific primers. The size of amplicons was ranged from 740 to 821 bp in length. The amplified polymerase chain reactions products were purified and were sequenced. Using an ABI automated sequencer, the sequencing was done for the selected amplicons by Chromous Biotech Pvt. Ltd., Bangalore, India. The forward and reverse sequences of each genotype were used to align the retrieved sequences for the target candidate gene. The identity of the candidate gene was further confirmed by the BLAST against the reference genome assembly of chickpea. The nucleotide sequences were analysed using the BLAST program from NCBI (www.ncbi.nlm.nih). The ORF finder in NCBI (http://www.ncbi.nlm.nih.gov/gorf/gorf.html) was used to identify the open reading frames (ORFs). The BioEdit software version 7.2.5 was used to align the sequenced DNA samples. The detection of SNPs (Single nucleotide polymorphisms) and mutations/deletions was also done by BioEdit. The present study validates the presence of a single band of *TPS* gene with the expected size in all the samples. Samples were subjected to sequencing for further confirmation.

### Computational analysis of *TPS* protein

Conserved motifs in *TPS* proteins were analyzed by the software MEME/TOMTOM (http://meme.sdsc.edu/meme/cgi-bin/meme.cgo). The default parameters of the input file for the MEME program were maintained in order of sequences in the phylogenetic tree to facilitate the observation of common motifs between the closely related sequences. The different properties of *TPS* protein genes for molecular weight (MW), isoelectric point (pI), and grand average of hydropathicity (GRAVY) were calculated by the software ProParam (http://web.expasy.org/protparam). The conserved domains of the *TPS* protein sequences were identified from the Conserved Domain Database of NCBI (CDD, www.ncbi.nlm.nih.gov/Structure/cdd/cdd.shtml). The transmembrane structure domain and Hydrophobic/hydrophilic features of protein were analyzed using TMpred (https://embnet.vital-it.ch/cgi-bin/TMPREDformparser). The RCSB/PDB, a homology model of protein database was used for predicting the 3D structure of target protein (https://www.rcsb.org/structure and validated with PDBsum tool)^29^. To determine the anticipated protein's functional and physical interactions, the STRING database (http://string-db.org/) was used to perform network analysis.

### Ethics statement

Collection of plant material, complies with all the institutional, national, and international guidelines and legislation.

## Supplementary Information

Supplementary Information.

## Data Availability

All the data of the present study are available within the manuscript, supplementary materials and in the NCBI data base with IDs: MF503402, MF503403, MF503405, MF503406, MF503407 and KY542279.
