# Supplementary material for: A comprehensive analysis of Trehalose-6-phosphate synthase (TPS) gene for salinity tolerance in chickpea (Cicer arietinum L.)
Source: Sci Rep. 2022 Sep 29;12:16315. doi: 10.1038/s41598-022-20771-x (PMC9523030; doi:10.1038/s41598-022-20771-x)
Supplement: Supplementary file 1 — Supplementary Information. [file 41598_2022_20771_MOESM1_ESM.doc]

**Supplementary Tables**

**Supplementary Table 1.** Fifty diverse genotypes containing varieties, germplasm collection, landraces, and wild derivatives.

| **S. NO.** | **VARIETY** | **TYPE** | **PEDIGREE/SOURCE** |
| --- | --- | --- | --- |
| **1** | CSG 8962 | Desi | Selection from GPF 7035 |
| **2** | JG 62 | Desi | Local selection from west Nimar (M.P.) |
| **3** | PUSA 1103 | Desi | (Pusa256 x C.reticulatum) x Pusa 362 |
| **4** | AVARODHI | Desi | (T-3 x K 315) |
| **5** | BGD 112 | Desi | (BG 209 x GL 84038) x Pusa 212 |
| **6** | ANNEGIRI | Desi | Local selection from germplasm of Karanataka |
| **7** | SBD 377 | Desi | ICCV 88109 x PRR 1) x ICC 4958 |
| **8** | PUSA 362 | Desi | (BG 203 x P 179) x BC 203 |
| **9** | ICC 1882 | Desi | Traditional landrace P1506-4 from ICRISAT |
| **10** | ICC 4958 | Desi | GW 5/7, a drought tolerant breeding line from ICRISAT |
| **11** | PUSA 547 | Desi | Mutant of BG 256 |
| **12** | PUSA 72 | Desi | (Pusa 256 x E 100Ym) x (Pusa 256) |
| **13** | ICCV 97119 | Desi | (avarodhixJG 62)X ICC 11551 |
| **14** | ICCV 06101 | Desi | ICC 5619 x ICC 37 |
| **15** | ICCV 00104 | Desi | JG 74 x ICCL 83105 |
| **16** | ICCV 10111 | Desi | ICCV 93954 x ICC5683 |
| **17** | L 550 | Kabuli | Pb7 x Rabat |
| **18** | ICCV 10 | Desi | P1231 × P1265 |
| **19** | ICCV 2 | Kabuli | F3 [(K 850 x GW 5/7) x P 458] x F3  (L 550x Guamuchil)-2 |
| **20** | GOKCEE | Kabuli | (Pusa 256 x ICCV 32) x ICCV 32 |
| **21** | ICCV 92337 | Kabuli | (ICCV 2x surutato 77) X ICC 7344 |
| **22** | ICCV 10316 | Kabuli | ICCV 92337 X ICC 17109 |
| **23** | ICCV 03302 | Kabuli | (L550 X ICC 14196)X ICCV 92392 |
| **24** | ICCV 01318 | Kabuli | ICCV 95311 X ICC 14194 |
| **25** | IG 5844a | Kabuli | Chickpea Landrace from Amman, Jordan |
| **26** | IG 5856 | Kabuli | Chickpea Landrace from Maan, Jordan |
| **27** | IG 5857 | Kabuli | Chickpea Landrace from Maan, Jordan |
| **28** | IG 5884 | Kabuli | Chickpea Landrace from Ninawa, Iraq |
| **29** | IG 5894 | Kabuli | Chickpea Landrace from Arbil, Iraq |
| **30** | IG 5906 | Kabuli | Chickpea Landrace from As Sulaymaniyah, Iraq |
| **31** | ILC0 (Italy) | Kabuli | Chickpea Landrace from Italy |
| **32** | ILC 10768 | Desi | Chickpea Landrace from Armenia |
| **33** | ILC0 (Czech Rep.) | Desi | Chickpea Landrace from Czech Republic |
| **34** | ILC0 (Syria) | Desi | Chickpea Landrace from Icarda Syria |
| **35** | ILC 8666 | Kabuli | Chickpea Landrace from Portugal |
| **36** | ILC0 (Latvia) | Desi | Chickpea Landrace from Latvia |
| **37** | ILC 1312 | Kabuli | Chickpea Landrace from Cyprus |
| **38** | IG 5855 | Kabuli | Chickpea Landrace from Jordan |
| **39** | IG 5867 | Kabuli | Chickpea Landrace from Jordan |
| **40** | IG 5890 | Kabuli | Chickpea Landrace from Iraq |
| **41** | IG 5895 | Kabuli | Chickpea Landrace from Iraq |
| **42** | IG 5896 | Kabuli | Chickpea Landrace from Iraq |
| **43** | IG 5904 | Kabuli | Chickpea Landrace from Iraq |
| **44** | IG 5980 | Kabuli | Chickpea Landrace from Spain |
| **45** | IG 5982 | Kabuli | Chickpea Landrace from Spain |
| **46** | IG 5985 | Kabuli | Chickpea Landrace from Spain |
| **47** | IG 6000 | Kabuli | Chickpea Landrace from Tunishia |
| **48** | GLW 91 | Desi | ICCV96030 x C. pinnatifidum acc.188 |
| **49** | GLW 69 | Desi | ICCV96030 x C. pinnatifidum acc.188 |
| **50** | GLW 36 | Desi | ICCV96030 x C. pinnatifidum acc.188 |

Supplementary Table 2. Subset of tolerant genotypes was selected for the validation of markers linked to candidate genes.

| **S. No.** | **Gene** | **Genotypes** | **Amplified Product**  **(bp)** | **Accession No.** |
| --- | --- | --- | --- | --- |
| **1** | **TPS** | JG 62 | 812 | MF503402 |
| **2** |  | PUSA 1103 | 740 | MF503403 |
| **3** |  | ICCV 10 | 752 | MF503405 |
| **4** |  | ICCV 2 | 815 | MF503406 |
| **5** |  | IG 5856 | 814 | MF503407 |
| **6** |  | PUSA 362 | 821 | KY542279 |

**Supplementary Table 3.** The gene sequence from the PUSA1103 genotype has shown the highest identity (99%) with e-value 5e-156 with the *TPS* gene (XM_004503283).

| **Gene** | **Amplicon**  **size (bp)** | **BlastN result** | **Accession No.** | **e-value** | **BlastX result** | **Accession No.** | **e-value** |
| --- | --- | --- | --- | --- | --- | --- | --- |
| **TPS**^a^ | 820 | Cicer arietinum alpha, alpha-trehalose-phosphate synthase [UDP-forming] 6 | XM_004503283 | 0.00***** | [alpha, alpha-trehalose-phosphate synthase [UDP-forming] 6 [Cicer arietinum]](https://blast.ncbi.nlm.nih.gov/Blast.cgi#alnHdr_502138256) | [XP_004503340.1](https://www.ncbi.nlm.nih.gov/protein/XP_004503340.1?report=genbank&log$=prottop&blast_rank=10&RID=HWETCN9H015) | 5e-156 |

**^a^ = Trehalose-phosphate synthase (TPS); * = 0.00 = <1e-179**

| **Motifs** | **Name** | **Functions** |
| --- | --- | --- |
| **Motif 1** | MADS-box  TFs AGL25 | Minichromosome maintenance1 Agamous Deficiens Serum response factors-MADS-box TFs AGL25 (AGAMOUS-Like25: involved in seed germination by influencing the ABA catabolic pathway and regulation ABA signaling |
|  | AGL63 | AGAMOUS-Like63: expressed in seeds and embryos; growth and Transcription regulation |
|  | AGL15 | AGAMOUS-Like15: expressed in most mature region: phloem procambium, elongation/maturation zone, meristematic zone) |
|  | AGL6 | AGAMOUS-Like6: highly expressed in Procambium) |
|  | AGL13 | AGAMOUS-Like13; highly expressed in Lateral root cap |
|  | HSP | Heat Shock transcription factor like Protein (HSFC1, HSFA1E, HSFA6B, HSFA6A, HSF7, HSF3, HSF6 and HSF21) |
|  | AP2/EREBP (CBF4) | APETALA2/ethylene-responsive element-binding protein (AP2/EREBP (CBF4): involved in cold and drought tolerance, responsive to ABA and regulate drought adaption |
|  | BSD | BSD domain containing protein (ATIG10720): a novel transcription factor which functions as a transcriptional activator |
| **Motif 2** | ARF2 | ARF2 (Auxin response factor 2) transcription factor, play an important role in auxin signaling, plant growth, development and stress response. ARF2 may affect seed size and drought tolerance through regulating ABA signaling |
|  | C2H2 | Cys2/His2 (C2H2) type zinc finger proteins play an important role in abiotic stress resistance in plant |
|  | bZIP52 | Basic leucine zipper (bZIP52) gene-regulate metabolic reprogramming during stress |
| **Motif 3** | RWP-RK  (NLP7) | RWP-RK a putative DNA-binding domain, which was previously proposed in the primary structure of NIN, NIN-LIKE PROTEIN genes (NLP7) a regulatory protein associated with nitrogen assimilation |
|  | WRKY50 | a DNA binding proteins characterized by the presence of the peptide sequence (Trp-Arg-Lys-Tyr) followed by a Zn-finger domain. They are important for stress induced transcriptional reprogramming |
|  | NAC (ANAC094) | NAC domain containing protein 94 function in transcription factor activity and involved in regulation of transcription. |
| **Motif 4** | NLP (AtNLP4) | NIN-LIKE PROTEIN genes (NLP4) is a regulatory protein associated with nitrogen assimilation |
|  | bZIP28 | Basic leucine zipper (bZIP52) gene-regulate metabolic reprogramming during stress |
|  | HSF | Heat Shock transcription factor like Protein |
| **Motif 5** | MYB (MYB96 and MYB 94) | these transcription factors have been found to be involved in the drought response |
|  | HMG(3xHMG-box1) | HMG (High mobility group)-AT4G11080 (3xHMG-box1) proteins are abundant chromatin-associated proteins found in nuclei which interact with mitotic and meiotic chromosomes |

**Supplementary Table 4** DNA motif show that the query motif closely resembles the binding motif for orthologous transcription factor proteins in arabidopsis.

**Supplementary Figures**

**
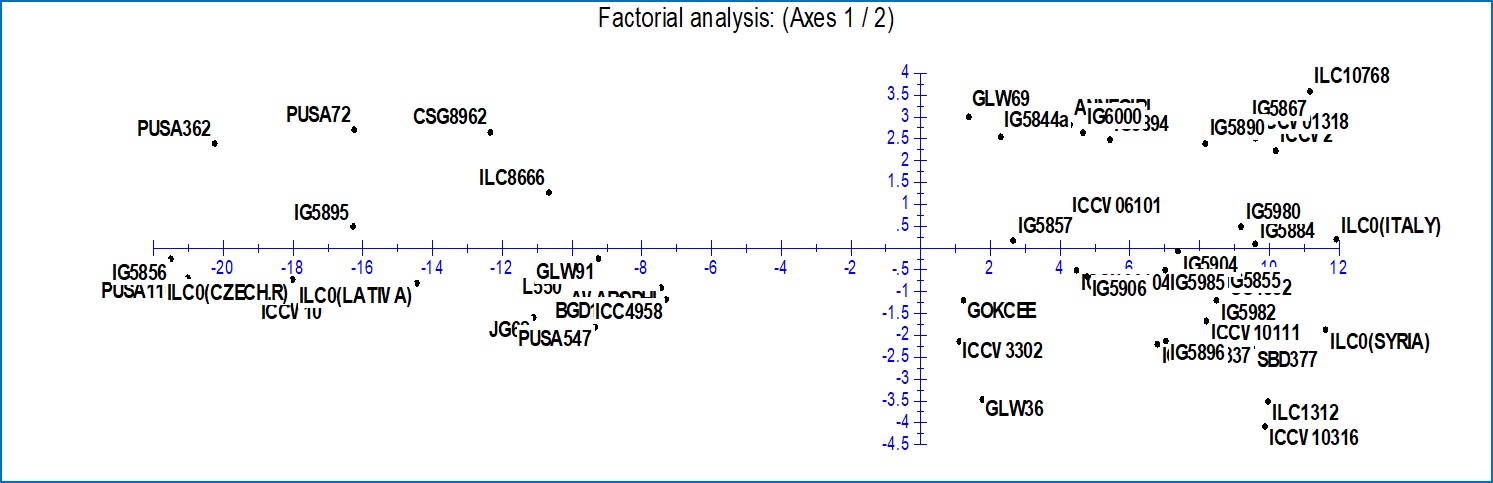
**

**Supplementary Figure 1.** A 2D plot of factorial analysis from the morphological traits to estimate the genetic variation in the genotypes. Scattered plot revealed a pattern of mostly two groups which were distinctively separated the tolerant (left-axis) and susceptible genotypes (right-axis).


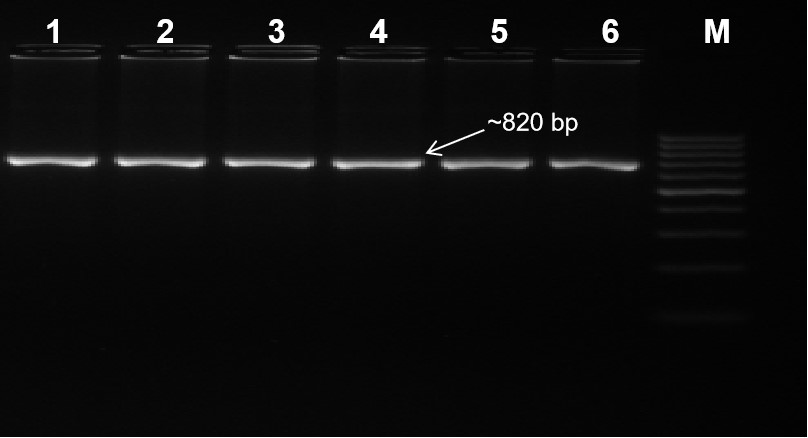


**Supplementary Figure 2.** *TPS* amplicons from selected genotypes (Lane:1-6) as in S.Table 2. Marker (M): 1 kb DNA ladder. The TPS was amplified using the gene-specific primers and the size of amplicon was ranged from 740-821 bp in length.


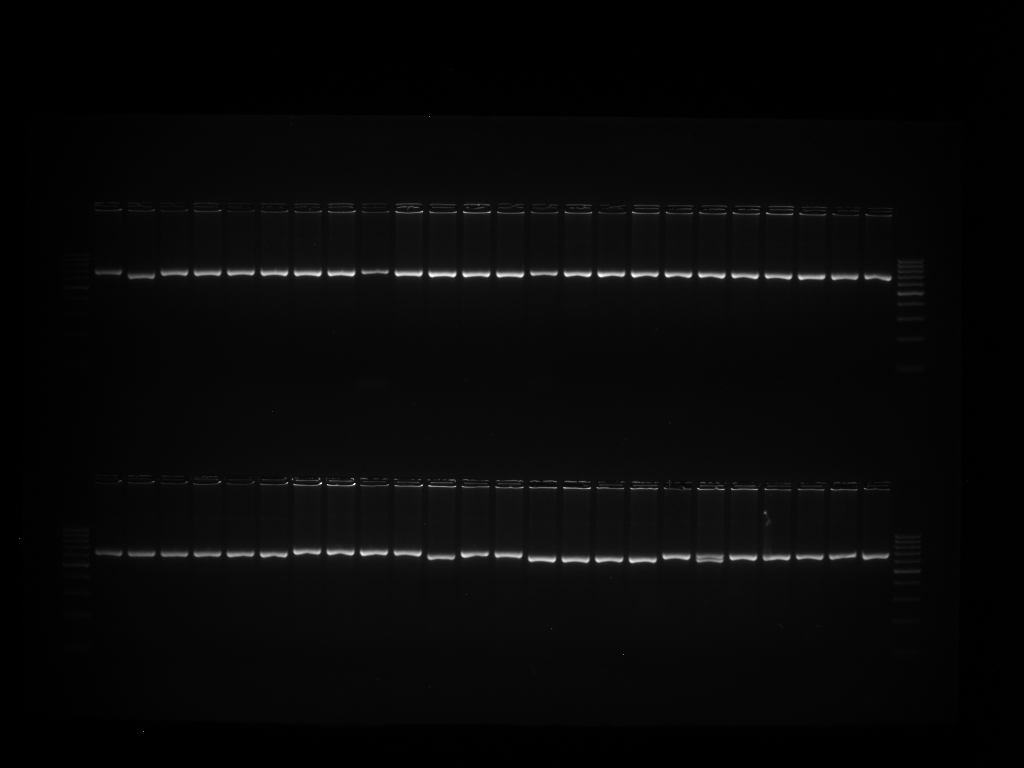


**Unedited gel image of Supplementary Fig.2**
